# Comparing the current short-term cancer incidence prediction models in Brazil with state-of-the-art time-series models

**DOI:** 10.1038/s41598-024-55230-2

**Published:** 2024-02-25

**Authors:** Daniel Bouzon Nagem Assad, Patricia Gomes Ferreira da Costa, Thaís Spiegel, Javier Cara, Miguel Ortega-Mier, Alfredo Monteiro Scaff

**Affiliations:** 1 Department of Industrial Engineering, Universidade do Estado do Rio de Janeiro, São Francisco Xavier, 524, Rio de Janeiro, Rio de Janeiro 20550-900 Brazil; 2 Escuela Técnica Superior de Ingenieros Industriales, Universidad Politécnica De Madrid, Jose Gutierrez Abascal, 2, 28006 Madrid, Madrid Spain; 3 Fundação Ary Frauzino para Pesquisa e Controle do Câncer, Inválidos, 212, Rio de Janeiro, Rio de Janeiro 20231-048 Brazil

**Keywords:** Oncology, Engineering, Mathematics and computing, Computational models, Machine learning, Statistical methods

## Abstract

The World Health Organization has highlighted that cancer was the second-highest cause of death in 2019. This research aims to present the current forecasting techniques found in the literature, applied to predict time-series cancer incidence and then, compare these results with the current methodology adopted by the Instituto Nacional do Câncer (INCA) in Brazil. A set of univariate time-series approaches is proposed to aid decision-makers in monitoring and organizing cancer prevention and control actions. Additionally, this can guide oncological research towards more accurate estimates that align with the expected demand. Forecasting techniques were applied to real data from seven types of cancer in a Brazilian district. Each method was evaluated by comparing its fit with real data using the root mean square error, and we also assessed the quality of noise to identify biased models. Notably, three methods proposed in this research have never been applied to cancer prediction before. The data were collected from the INCA website, and the forecast methods were implemented using the R language. Conducting a literature review, it was possible to draw comparisons previous works worldwide to illustrate that cancer prediction is often focused on breast and lung cancers, typically utilizing a limited number of time-series models to find the best fit for each case. Additionally, in comparison to the current method applied in Brazil, it has been shown that employing more generalized forecast techniques can provide more reliable predictions. By evaluating the noise in the current method, this research shown that the existing prediction model is biased toward two of the studied cancers Comparing error results between the mentioned approaches and the current technique, it has been shown that the current method applied by INCA underperforms in six out of seven types of cancer tested. Moreover, this research identified that the current method can produce a biased prediction for two of the seven cancers evaluated. Therefore, it is suggested that the methods evaluated in this work should be integrated into the INCA cancer forecast methodology to provide reliable predictions for Brazilian healthcare professionals, decision-makers, and oncological researchers.

## Theoretical background

A time series is a sequence of time-oriented observations related to forecasting or controlling a specific variable^1^. This thematic study originated in 1927, adopting a general approach to time series analysis^2^. Nearly three decades later, new time series forecasting approaches began to emerge.

Initially, classical time series statistical models were proposed^3^. Subsequently, these models were refined to include exponential smoothing techniques^4,5^ before evolving into auto-regressive moving average models^6^. Eventually, they progressed further to incorporate Machine Learning^7^ and State-Space models^8^.

In all instances, the predictability of future events is a central element, crucial for planning and processes related to Operations Management, among others, such as Marketing, Economics, and Demography^1^. However, the predictability of an event or quantity depends on various factors, including an understanding of the influencing factors, data availability, future and past similarities, and the potential impact of forecasts on the predicted outcome^9^.

In the context of oncology studies, mortality and incidence projection methods were already compared in Canada, using age-period-cohort (APC), auto-regressive time series, and space-state models at least for ten cancer types^10^.

APC and Bayesian APC, auto-regressive integrated moving average (ARIMA) time series, and simple linear models were also compared for five cancer types in Switzerland^11^.

Using reported breast cancer cases in the Fijian population from 1995 to 2016, Chand et al.^12^ attempted to apply an ARIMA model to provide a 12-month ahead prediction. However, faced with non-stationary data according to the Augmented Dickey-Fuller test, a linear regression model was chosen. The proposed model was compared with the Naive Forecast Method, showing that the linear regression model outperformed the Naive Forecast Method.

Also exploring the epidemiological characteristics of breast cancer, Lin et al.^13^ used Exponential Smoothing (ETS) and Autoregressive Integrated Moving Average (ARIMA) models to forecast breast cancer incidence in China.

Regarding palliative cancer care, two different long short-term memory (LSTM) models were proposed, aiming to forecast the patients’ next visit day and estimate the total patient demand 1 week ahead^14^. For this, was take into account their requirements, demographics, and each service history profile.

Alrobai and Jilani^15^ also applied LSTM to forecast the incidence of the three most prevalent cancers in Saudi Arabia. However, it’s crucial to note that cancer prevalence can significantly vary from one country to another.

In Malaysia, to deal with the continued annual growth in cancer incidence rates, particularly female breast, colorectal, and lung cancer, Lazam et al.^16^ tested ARIMA and Exponential Smoothing (ETS) models. They intended to determine the best rates for incidence prediction for these mentioned types of cancer.

Tudor^17^ proposed alternative ways to forecast cancer incidence and mortality by connecting population web-search practices with health variables officially published by Romanian authorities. The applied models included ARIMA, the Exponential Smoothing State-Space Model with Box-Cox Transformation, ARMA Errors, Trend, and Seasonal Components, and a feed-forward neural network nonlinear autoregression model.

In this research, conducted in Brazil, we present the framework to evaluate previous works on cancer time-series prediction, dividing the time-series prediction according to Hyndman and Athanasopoulos^9^ into Classical Statistical models, State-Space models, and Machine Learning models (Table 1). For this, only researches that makes cancer predictions were considered.

**Table 1:** Forecasting model applied by cancer type. *Source*: The authors.

Forecasting approaches	CSM		SSM		MLM		
Model classes	ETS	ARIMA	TBATS	KF	NNETAR	MLP	LTSM
Breast cancer	^13,16,18^	^10–13,16,19^		^10^			^14^
Colorectal cancer	^16^	^11,16,19^					^14,15^
Prostate cancer		^10,11,19^		^10^			^14^
Lung cancer	^16^	^10,11,16,19,20^		^10^			^14^
Cervical cancer		^10,19^		^10^			^14^
Head and Neck cancer		^10,19^		^10^			^14^
Childhood cancer		^19^					^14^
Skin melanoma and others		^10,11,19^		^10^			^14^

After comparing ten previous works related to cancer incidence prediction (Table 1), we can conclude that:

1. Breast and lung cancer incidence predictions have garnered more attention in specialized literature and have been studied in 8 and 7 works, respectively; colorectal cancer has been studied in 5 works, while other cancer types have been studied in 4 works or less.
2. CSM and particularly ARIMA were the most used approaches.
3. Considering SSM and MLM, TBATS NNETAR, and MLP were never covered before in previous research.
4. We found no previous work in which all three classes of models were applied.

As will be presented in this paper, the third and fourth conclusions allow us to state that this work covers a gap in current cancer prediction. Thus, applying unseen methods (3rd) and the three classes of models (4th) to cancer prediction is an **original contribution** of this research.

Finally, the mentioned studies address the application of different forecasting methods in countries such as Canada, Switzerland, Fiji, China, Malaysia, and Romania. Their use in Brazil, for a larger sample of types of cancer and comparing them, seems like a complementary contribution.

## Methods

### Data collection

**Figure 1:**
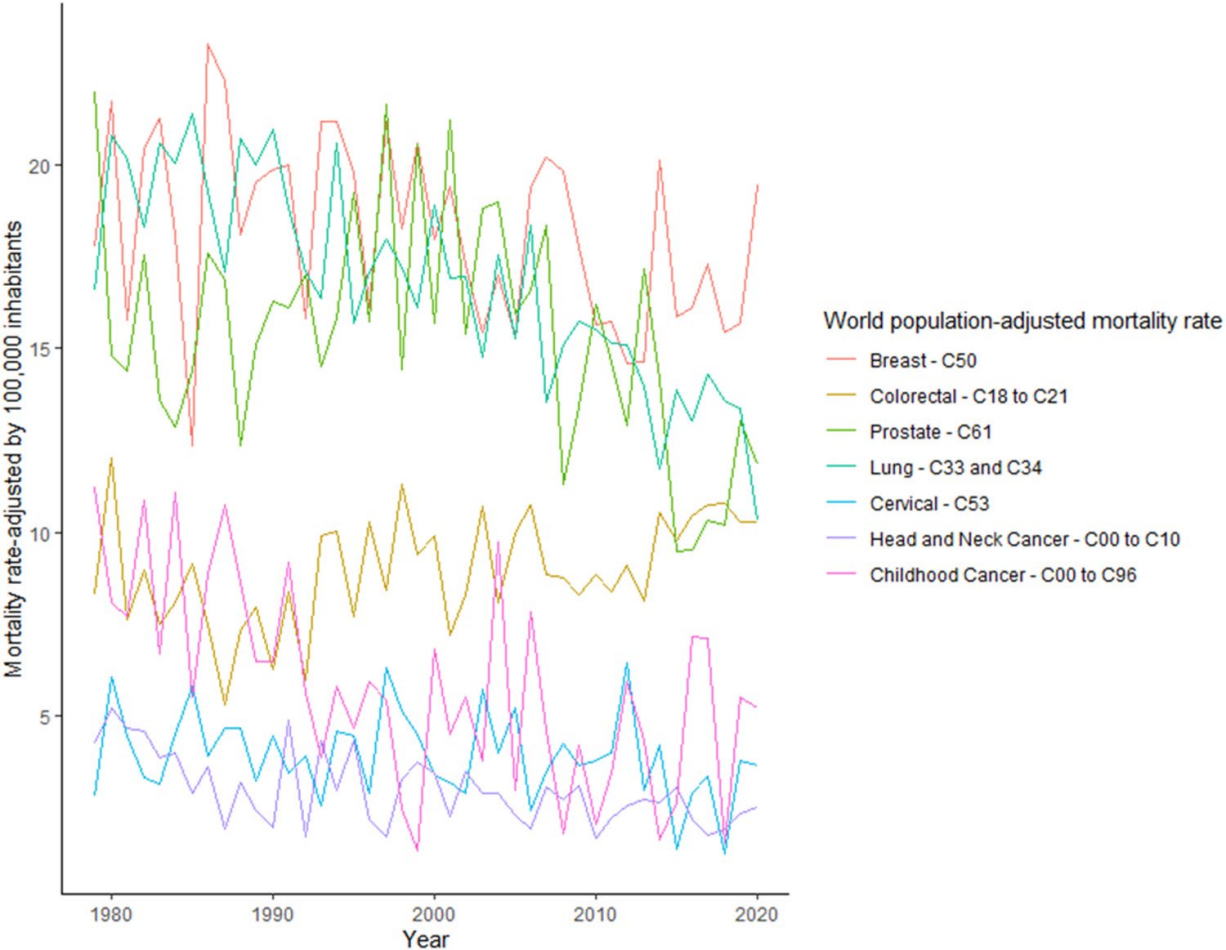
ICD-10 Mortality rate by 100,000 inhabitants considering world population-adjusted by cancer type.

In this research, we analyze real cancer data from Brazil obtained from INCA. All time-series used are presented in Fig.  1 and are also available at Table 2. The seven cancer types evaluated are: Breast cancer (ICD-10 C50), Colorectal Cancer (ICD-10 C18 to C21), Prostate cancer (ICD-10 C61), Lung cancer (ICD-10 C33 and C34), Cervical cancer (ICD-10 C53), Head and Neck Cancer (ICD-10 C00 to C10) and Childhood Cancer (ICD-10 C00 to C96).

**Table 2:** ICD-10 Mortality rate by 100,000 inhabitants considering world population-adjusted by cancer type.

Year	Breast	Colorectal	Prostate	Lung	Cervical	Head and Neck	Childhood
1979	17.77	8.30	21.95	16.59	2.84	4.25	11.20
1980	21.73	12.03	14.81	20.81	6.09	5.23	8.10
1981	15.79	7.64	14.39	20.17	4.49	4.67	7.72
1982	20.43	8.97	17.53	18.31	3.32	4.60	10.86
1983	21.27	7.50	13.59	20.57	3.17	3.87	6.72
1984	18.08	8.08	12.88	20.02	4.52	3.99	11.09
1985	12.37	9.14	14.38	21.39	5.80	2.91	5.54
1986	23.27	7.52	17.59	19.29	3.91	3.62	8.84
1987	22.28	5.30	16.87	17.06	4.68	1.94	10.76
1988	18.08	7.34	12.37	20.69	4.70	3.22	8.66
1989	19.53	7.97	15.11	19.97	3.25	2.44	6.48
1990	19.86	6.29	16.29	20.97	4.47	1.98	6.49
1991	19.99	8.38	16.11	18.79	3.46	4.90	9.19
1992	15.82	6.00	17.01	17.06	3.94	1.72	5.65
1993	21.17	9.92	14.49	16.37	2.58	4.33	3.88
1994	21.17	10.02	15.83	20.59	4.61	3.00	5.81
1995	19.79	7.72	19.21	15.70	4.45	4.36	4.68
1996	16.11	10.28	15.72	17.09	2.93	2.19	5.95
1997	21.17	8.43	21.62	17.98	6.33	1.73	5.46
1998	18.26	11.31	14.44	17.21	5.15	3.28	2.50
1999	20.59	9.41	20.55	16.09	4.51	3.74	1.37
2000	17.97	9.89	15.70	18.91	3.41	3.45	6.83
2001	19.39	7.20	21.21	16.92	3.22	2.29	4.51
2002	17.31	8.29	15.39	16.97	2.91	3.51	5.52
2003	15.44	10.71	18.82	14.76	5.73	2.92	3.80
2004	17.00	8.09	18.97	17.53	3.99	2.92	9.74
2005	15.30	10.00	15.96	15.25	5.21	2.32	2.99
2006	19.39	10.77	16.59	18.33	2.46	1.94	7.86
2007	20.18	8.84	18.35	13.52	3.49	3.06	4.63
2008	19.81	8.78	11.32	15.11	4.24	2.73	1.82
2009	17.77	8.31	13.42	15.73	3.66	3.12	4.22
2010	15.64	8.84	16.20	15.50	3.81	1.67	2.05
2011	15.75	8.38	14.65	15.12	3.99	2.22	3.47
2012	14.58	9.12	12.91	15.08	6.45	2.56	5.95
2013	14.63	8.15	17.17	13.95	3.00	2.72	4.35
2014	20.11	10.54	14.18	11.71	4.21	2.67	1.65
2015	15.86	9.80	9.50	13.87	1.38	3.08	2.66
2016	16.09	10.47	9.54	13.01	2.92	2.19	7.15
2017	17.27	10.75	10.34	14.29	3.39	1.78	7.11
2018	15.45	10.80	10.21	13.58	1.27	1.96	1.58
2019	15.70	10.29	13.04	13.35	3.79	2.35	5.51
2020	19.46	10.31	11.86	10.33	3.68	2.52	5.23

The filters employed for each type of cancer can be found in Table 3. We gathered data on the mortality rates for Brazilian cancer from INCA’s website^21^. The population figures were obtained from the 2022 Brazilian census^22^.

**Table 3:** Filters and criteria used to retrieve cancer data by cancer type.

Cancer type	ICD-10	Gender	Age (in years)	District	Population
Breast cancer	C50	Female	All	Niterói	276,362
Colorectal cancer	C18 to C21	All	All	Niterói	508,470
Prostate cancer	C61	Male	All	Niterói	232,108
Lung cancer	C33, C34	All	All	Niterói	508,470
Cervical cancer	C53	Female	All	Niterói	276,362
Head and Neck cancer	C00 to C10	All	All	Niterói	508,470
Childhood cancer	C00 to C96	All	0 to 19	Niterói	105,930

In Brazil, the cancer incidence is not registered to all districts. So, INCA works with an approximate incidence inferred from the mortality rate considering Black et al.^23^, Ferlay et al.^24^ and Ferlay et al.^25^ estimation methodologies based mainly on the I/M ratio.

The mentioned methodologies links the unknown incidence rate-adjusted (*IRa*) to the known mortality rate-adjusted (*MRa*) of some district by the equation $IRa = MRa*(I_R/M_0)$. Where $I_R$ refers to known incidence of districts geographically near from the targeted unmeasured district and $M_0$ refers to number of deaths of the same districts. The results of $I_R/M_0$ ratio to the unmeasured district evaluated is presented in Table 4.

**Table 4:** I/M ratio by cancer type.

Type of cancers	$I_R/M_0$ ratio
Breast	4.37
Colorectal	2.4
Prostate	5.59
Lung	1.03
Cervical	2.69
Head and Neck	2.27
Childhood	2

In Fig.  1 we present for each type of studied cancer the mortality rate by world population-adjusted by 100,000 inhabitants. Then, according to^23−25^ estimation methodologies the expected incidence rate-adjusted *IRa* can be obtained by multiplying the mortality rate-adjusted in Fig.  1 and the values presented in Table 4 to each cancer type.

The current short-term predictions in Brazil rely on the average of the past 3 recent years. This outcome serves as a reference for the Brazilian public health system over the next 3 years. In essence, the existing approach is a simple moving average (MA).

### Forecasting models applied

In this research we apply the univariate forecasting methods available in Hyndman and Khandakar^26^, Petris^27^ and Kourentzes^28^. Models applied in next sections are presented in Table 5. These models were implemented in R^29^ language (version 4.1.3) and the code used is available at Supplementary Material (Forecasting code.R).

**Table 5:** Forecasting models applied in this research.

Type of models	Models
Current	Incidence average of the last 3 years
CSM	ETS, ARIMA^26^
SSM	TBATS^26^, KF^27^
MLM	MLP^28^, NNETAR^26^

**Figure 2:**
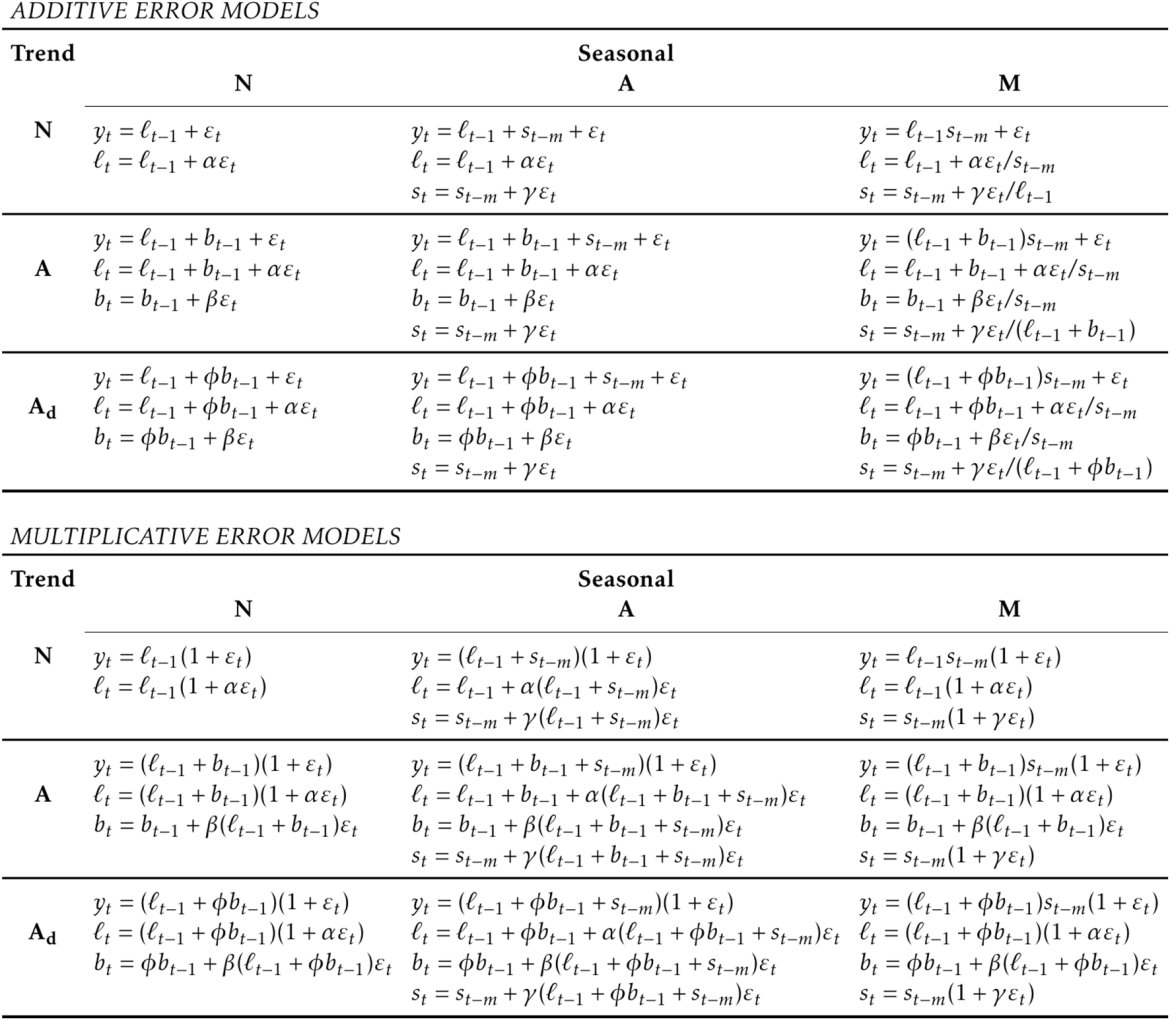
Hyndman and Athanasopoulos^9^ ETS equations.

To build each model is necessary to estimate many parameters, but the main features of each model are presented forward:

- ETS: ETS is a class of models that essentially works with three components equations level ($l_t$), trend ($b_t$) and season ($s_t$) to explain the original time series variable ($y_t$) that we aim to forecast. In each model these components cannot be significant, also known as None (N) or can be significant and better described $y_t$ as Additive (A) or Additive Damped (Ad) or Multiplicative (M) features. This class of models can be combined in 18 different ways (Fig.  2). For more details see Hyndman and Athanasopoulos^9^.
- ARIMA: ARIMA or Seasonal ARIMA (SARIMA) is a class of models that combine autoregressive (AR) and moving average (MA) with differenced values. The AR part of ARIMA (*p*) shows that the time series is regressed on its own past data. The MA part of ARIMA (*q*) indicates that the forecast error is a linear combination of past respective errors. The I part of ARIMA (*d*) refers to differenced values of *d* order to obtain stationary time-series in which ARMA model approach can be applied Kotu and Deshpande (2019)^30^. The difference between ARIMA and SARIMA models remains on the same components appearing lagged by the length of seasonal time window (frequency) as *P*, *D* and *Q*. For more details see Hyndman and Athanasopoulos^9^ and Kotu and Deshpande^30^.
- Kalman filter (KF): KF methods search the smallest vector that summarizes the past of the system that better describes the state of a deterministic dynamic system^31^. KF equation is basically composed by a linear autoregressive equation ${x(t)} = A*x(t) + W(t)$ where $W(t) \approx N(0,Q)$ with a measurement that is ${y(t)} = C*y(t) + V(t)$ where $V(t) \approx N(0,R)$ that defines the linearized process in which $y(t) \in {\mathbb {R}}$. The random variables *W*(*t*) and *V*(*t*) are assumed to be independent of each other and both must follow a normal distribution.
- TBATS: TBATS model is Trigonometric Seasonal (T) Exponential Smoothing Method + Box-Cox Transformation + ARMA model for residuals (BATS). Equations of the TBATS model are presented in equations below where $\omega$ and $\phi$ are Box-Cox and the damping parameters respectively, ARMA(*p*, *q*) process model the error and $m_1$ to $m_J$ list the seasonal periods used while $k_1$ to $k_J$ are the corresponding number of Fourier terms used. For more details see De Liveira et al.^32^.
  $$\begin{aligned} y_{t}^{(\omega )}= & {} \frac{ y_{t}^{(\omega )}-1}{\omega }, \omega \ne 0,\\ y_{t}^{(\omega )}= & {} \log {y_{t}}, \omega = 0,\\ y_{t}^{(\omega )}= & {} l_{t-1}+\phi *b_{t-1}+\sum _{i=1}^{t} s_{t-m_i}^{i} +d_t,\\ l_{t}= & {} l_{t-1}+\phi *b_{t-1} +\alpha *d_t,\\ b_{t}= & {} (1-\phi )*b_t +\phi *b_{t-1}+\beta *d_t,\\ s_{t}^{i}= & {} s_{t-m_i}^{i} +\gamma _i *d_t,\\ d_{t}= & {} \sum _{i=1}^{p} \phi _i*d_{t-i}+\sum _{i=1}^{q} \theta _i*\epsilon _{t-i} +\epsilon _{t},\\ s_{t}^{i}= & {} \sum _{j=1}^{k_j} s_{j,t}^{i},\\ s_{t}^{i}= & {} s_{j,t-1}^{i}*\cos {\lambda _j^i} + s_{j,t-1}^{*i}*\sin {\lambda _j^i} + \gamma _1^i*d_t,\\ s_{t}^{*i}= & {} s_{j,t-1}^{i}*\sin {\lambda _j^i} + s_{j,t-1}^{*i}*\cos {\lambda _j^i} + \gamma _2^i*d_t\\ \end{aligned}$$
- NNETAR: Neural Network Time Series Forecasts (NNETAR) is a class of feed-forward neural networks with a single hidden layer and lagged inputs. This model works with 2 (for non seasonal time-series) or 3 (for seasonal time-series) parameters: the number of past observations used as input layers (*p*), the number of past observations lagged by the length of seasonal time window used as input layers (*P*) and the number of neurons (*k*) in the single layer. In this research, a total of 20 repeats networks are fitted, each with random starting weights. These are then averaged when computing forecasts. The network is trained for one-step forecasting. Multi-step forecasts are computed recursively. The *k* selected to each type of cancer it the half of the number of input nodes plus 1. For non-seasonal data, the fitted model is denoted as an NNAR (*p*, *k*) (Neural Network Autoregressive) model which is analogous to an AR (*p*) model but with nonlinear functions. For seasonal data, the fitted model is called an NNAR (*p*, *P*, *k*)[*m*] model, which is analogous to an ARIMA (*p*, 0, 0)(*P*, 0, 0)[*m*] model but with nonlinear functions. For more details see Hyndman and Athanasopoulos^9^.
- MLP: MLP is an extension of feed-forward neural network where an arbitrary number of hidden layers that are placed in between the input and output layer (the truly computational engine of the MLP). According to Kourentzes et al.^33^, MLPs are designed to approximate any continuous function and can solve problems which are not linearly separable. In our case, the time-series problem proposed our input layer (like NNETARs’ model *p*) are the most recent past observations and we set the MLP model to choose the best number of input layers between 1 and the prediction length (3 years) lags will be used according to Mean Square Error. The same criteria were also adopted to choose the number of hidden nodes in each hidden layer. For more details see Kourentzes et al.^33^.

### Forecasting models evaluation

**Figure 3:**
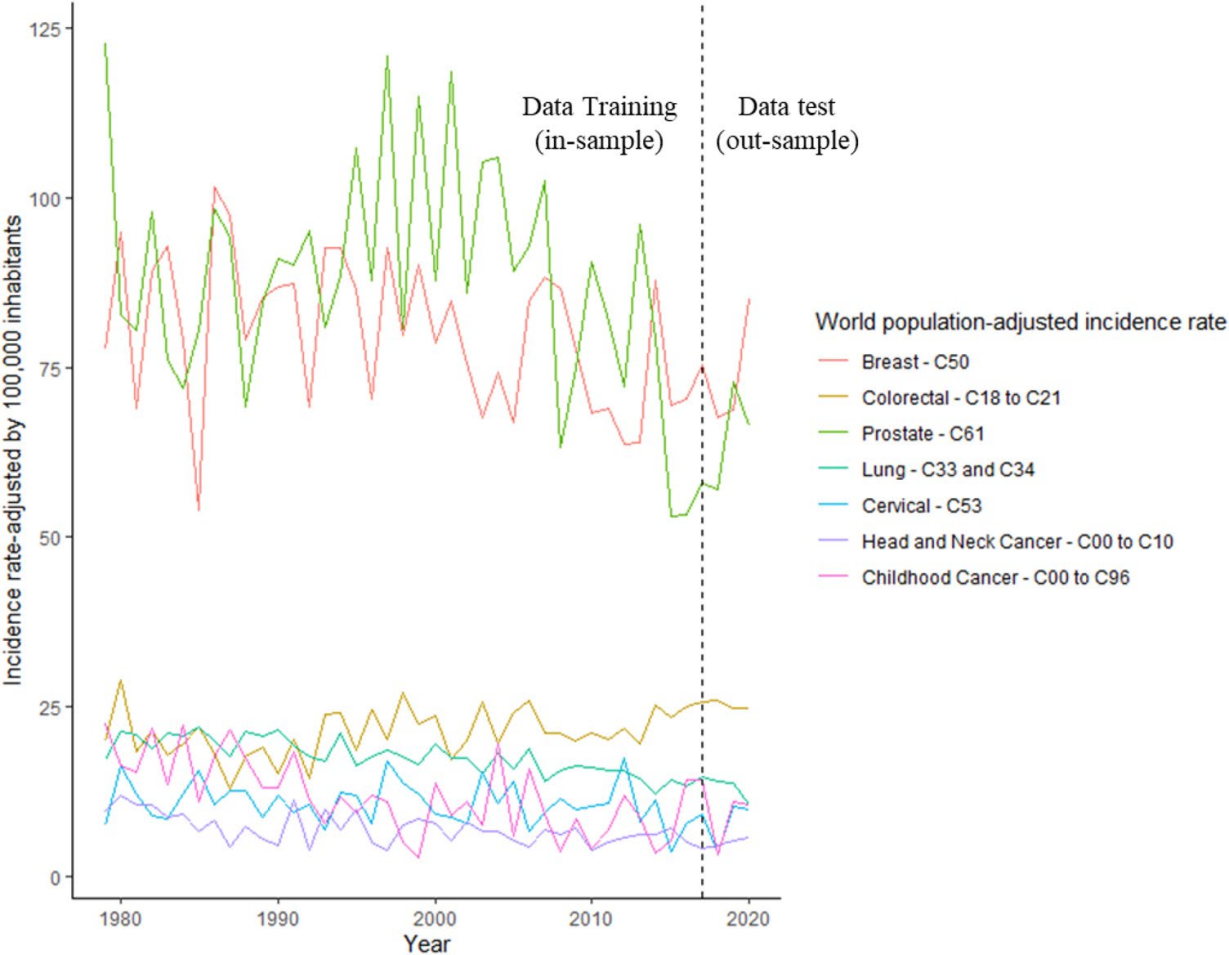
ICD-10 Incidence rate-ajusted (*IRa*) by cancer type.

The dataset presented in Table 2 were multiplied by I/M ratio for each cancer type shown in Table 4 to estimate the incidence rate of each type of cancer evaluated (Fig.  3).

For instance, to Breast cancer, the ICD-10 Mortality rate by 100,000 inhabitants are 17,77 in 1979, 21.73 in 1980 and so on (second column Table 2). Thus, the Breast cancer Incidence rate-ajusted will be these values multiplied by 5.59 (Breast cancers’ $I_R/M_0$ ratio in Table 4) which are 77.65 in 1979, 94.96 in 1980, 69 in 1981 and so on that can be seen in Fig.  3.

In this research, we are interested in provide a comparison between Brazilian’s current short-term cancer prediction and the time-series state of art models. As mentioned in Section Theoretical Background, as long as the current short-term cancer prediction are made 3 years ahead, we split our dataset into training data (from 1979 to 2017) and test data (from 2018 to 2020).

Training (in sample) and test (out of sample) data are evaluated using the Root Mean Square Error (RMSE) criterion. A low RMSE in sample value indicates a good average fit of the model used while a low value of RMSE out of sample indicates that the model used, on average, delivers a reliable forecast^9^.

Below we present the criteria adopted to evaluate the current and proposed methods predictions to each cancer type:

- The noise evaluation over the training (in sample) data according to the following tests: student (ST), normality (NT), Auto-correlation function (ACF) plot and Breusch-Pagan (BPT);
- The error evaluation according to the test (out of sample) Root Mean Square Error (RMSE).

If the residuals produced a 0 mean error in Student-test, follows a normal distribution in Shapiro–Wilk test, remains between the interval defined by the blue lines in ACF plot test to all lags and presented no constant variance all over the time (homoscedasticity) in Breusch-Pagan test, we consider that the model residuals produced a white noise which means that the model is unbiased^34–38^.

The significance level adopted in this research is 0.05 which means that residuals produced a white noise if the obtained *p*-values in each test are higher than 0.05 to each model.

Thus, in this research we consider that the best model for each cancer type is given by their residual evaluation that (1) fulfill all requirements previously presented and (2) obtained the lowest out of sample RMSE.

## Results

In this section we apply the methods presented in columns of Table 5 to each type of cancer incidence presented in Fig.  3. In Table 6 we summarize the in sample and out of sample RMSE results by model and type of cancer.

**Table 6:** RMSE per type of cancer per model.

RMSE	Model	Breast	Colorectal	Prostate	Lung	Cervical	Head and neck	Childhood
In Sample	Current	11.958	3.400	15.541	1.818	3.556	2.023	4.827
	ETS	10.686	3.375	15.418	1.541	3.099	1.675	4.114
	ARIMA	10.688	3.377	15.340	1.638	3.099	1.925	4.458
	TBATS	10.679	3.377	15.374	1.583	3.136	1.718	4.392
	KF	8.233	2.117	10.322	1.240	2.569	1.433	3.499
	NNETAR	10.588	2.504	10.889	1.500	2.999	1.366	3.213
	MLP	0.313	0.113	0.102	0.080	0.147	0.057	0.131
Out of Sample	Current	8.383	2.432	20.940	1.592	4.169	0.834	3.821
	ETS	8.128	0.956	6.489	1.490	4.172	0.856	3.666
	ARIMA	8.084	0.832	6.505	1.274	4.170	0.557	4.013
	TBATS	8.144	0.998	6.538	1.267	3.957	1.019	4.298
	KF	8.499	0.635	12.501	1.425	3.349	0.701	3.597
	NNETAR	9.684	1.119	14.284	1.814	3.929	1.293	4.702
	MLP	10.196	0.609	21.848	2.960	2.614	1.346	3.879

As mentioned in Forecasting models evaluation section, to compare models errors summarized in Table 6 we select the out of sample RMSE criterion. Then, to ensure that models residuals give us a white noise in the training data we apply the Student test (Table 7), the ACF plot, the Shappiro-Wink normality test (Table 8) and the Breusch-Pagan test (Table 9).

**Table 7:** Student test *p* value per type of cancer per model.

*p* values	Breast	Colorectal	Prostate	Lung	Cervical	Head and neck	Childhood
Current	0.811	0.819	0.352	0.386	0.759	0.387	0.677
ETS	0.249	0.489	0.325	0.285	0.998	0.933	0.890
ARIMA	0.384	0.684	0.729	0.259	1.000	0.124	0.189
TBATS	0.314	0.460	0.385	0.523	0.458	0.900	0.188
KF	0.226	0.229	0.677	$<0.05$	0.405	0.205	0.316
NNETAR	1.000	0.991	1.000	0.997	0.997	0.988	0.999
MLP	0.933	1.000	0.920	0.997	0.969	0.905	0.911

**Table 8:** Normality test *p* value per type of cancer per model.

p values	Breast	Colorectal	Prostate	Lung	Cervical	Head and neck	Childhood
Current	0.880	0.690	0.398	0.603	0.476	0.518	0.134
ETS	0.485	0.848	0.402	0.576	0.422	0.362	0.417
ARIMA	0.641	0.753	0.674	0.385	0.422	0.950	0.105
TBATS	0.558	0.864	0.639	0.723	0.438	0.501	0.125
KF	0.793	0.696	0.676	0.210	0.191	$<0.05$	0.203
NNETAR	0.385	0.912	0.773	0.509	0.999	0.079	0.257
MLP	$<0.05$	$<0.05$	$<0.05$	$<0.05$	$<0.05$	$<0.05$	$<0.05$

**Table 9:** Breusch—Pagan test *p* value per type of cancer per model.

*p* values	Breast	Colorectal	Prostate	Lung	Cervical	Head and neck	Childhood
Current	0.145	0.079	0.631	0.326	0.462	0.373	0.294
ETS	0.144	0.083	0.952	0.565	0.577	0.197	0.396
ARIMA	0.118	0.067	0.661	0.290	0.577	0.151	0.409
TBATS	0.105	0.083	0.492	0.299	0.569	0.033	0.513
KF	0.698	0.648	0.239	0.780	0.103	0.489	0.129
NNETAR	0.123	$<0.05$	0.306	0.221	0.182	0.368	0.406
MLP	0.944	$<0.05$	0.936	0.633	0.570	0.540	0.639

As mentioned in Section Forecasting models evaluation, besides considering RMSE criteria we must also evaluate if each model produced residual values with a white error noise taking into account their auto-correlation plots and normality test to all cancer types (Table 6).

This evaluation is presented for all types of cancer evaluated, grouped (Figure 4) and individually—breast (Figure 5), colorectal (Figure 6), prostate (Figure 7), lung (Figure 8), cervical (Figure 9), head and neck (Figure 10) and childhood (Figure 11).

**Figure 4:**
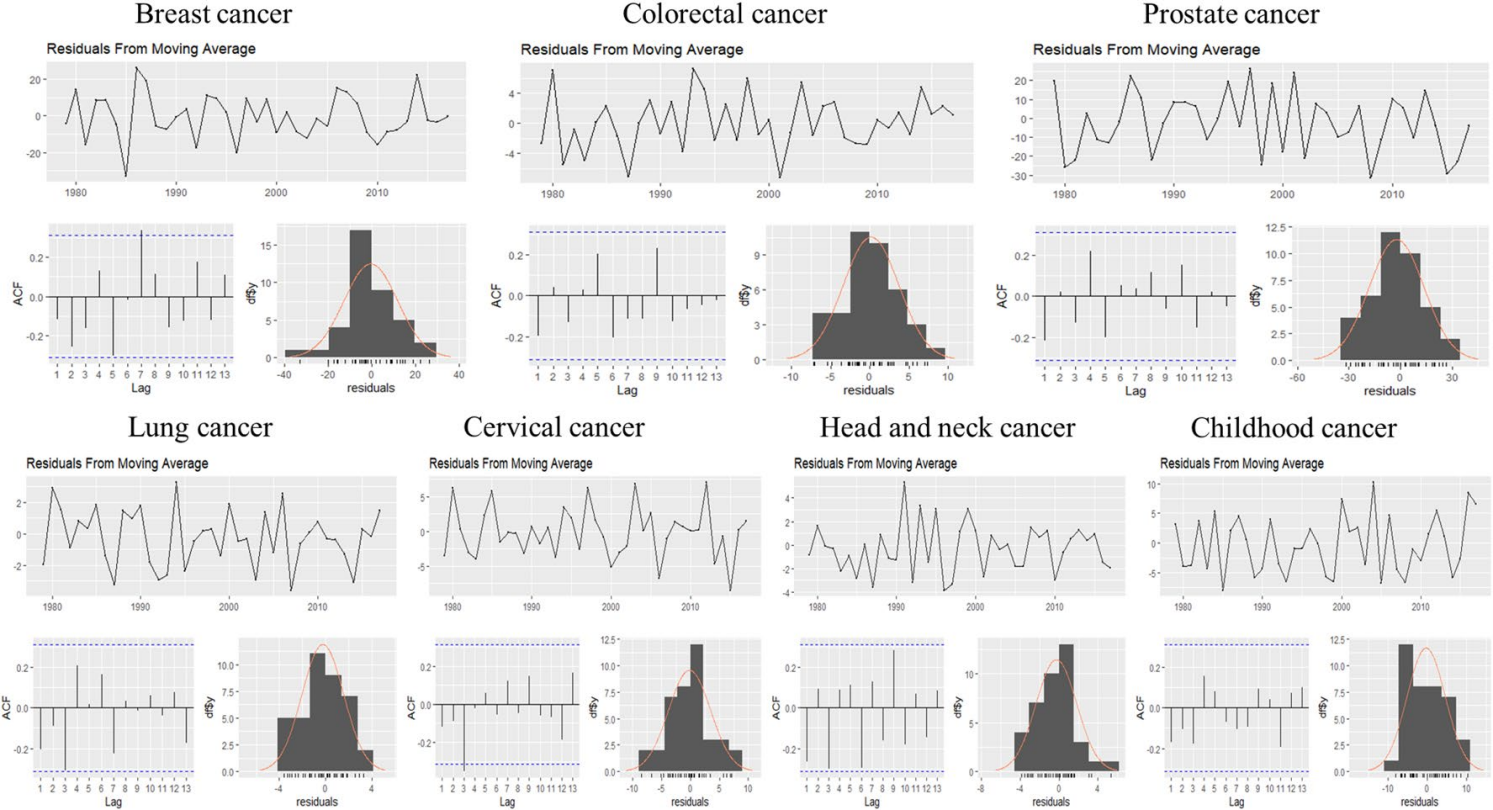
All cancer types noise evaluation using INCA’s current model.

**Figure 5:**
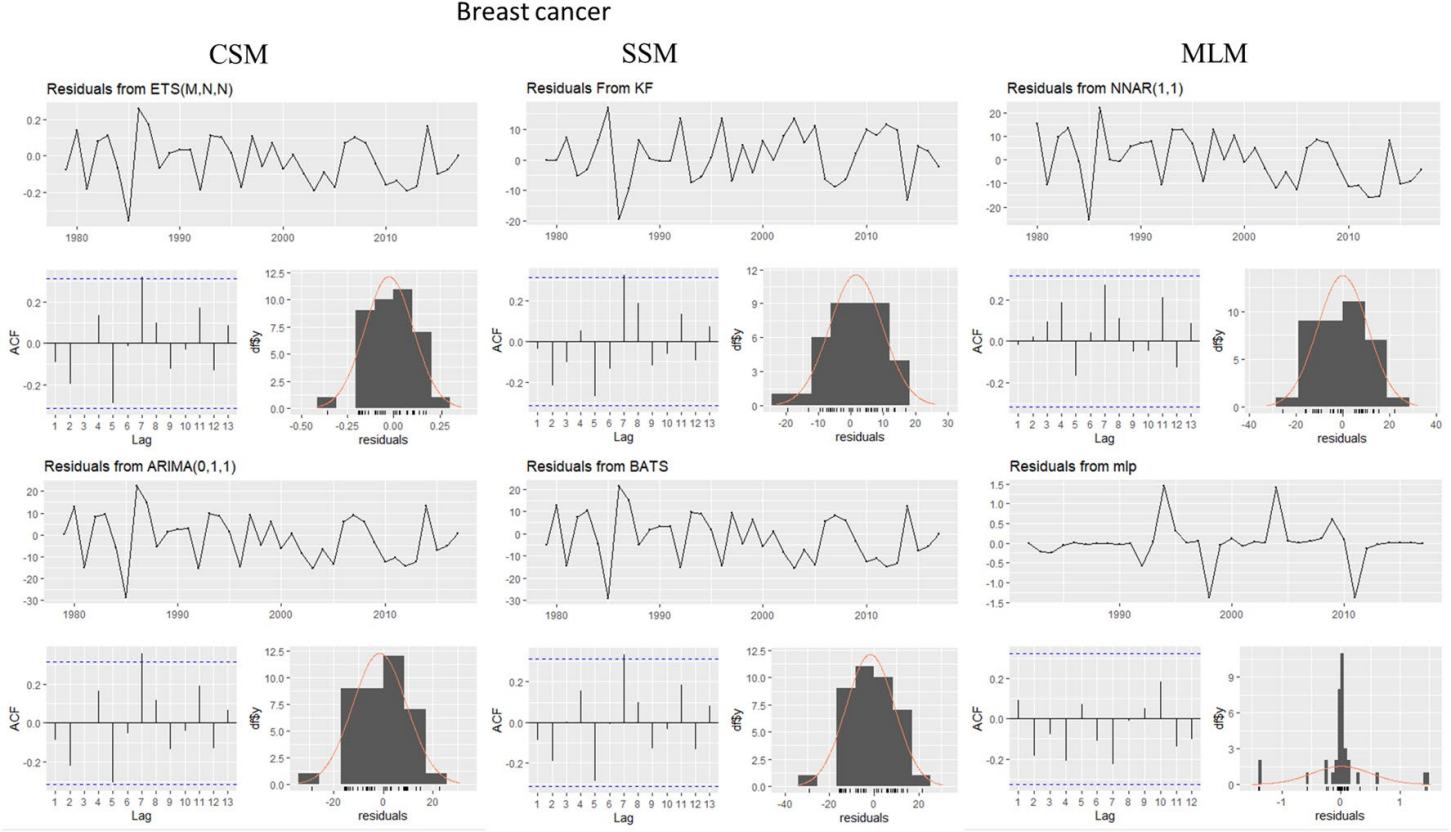
Breast cancer noise evaluation by model.

**Figure 6:**
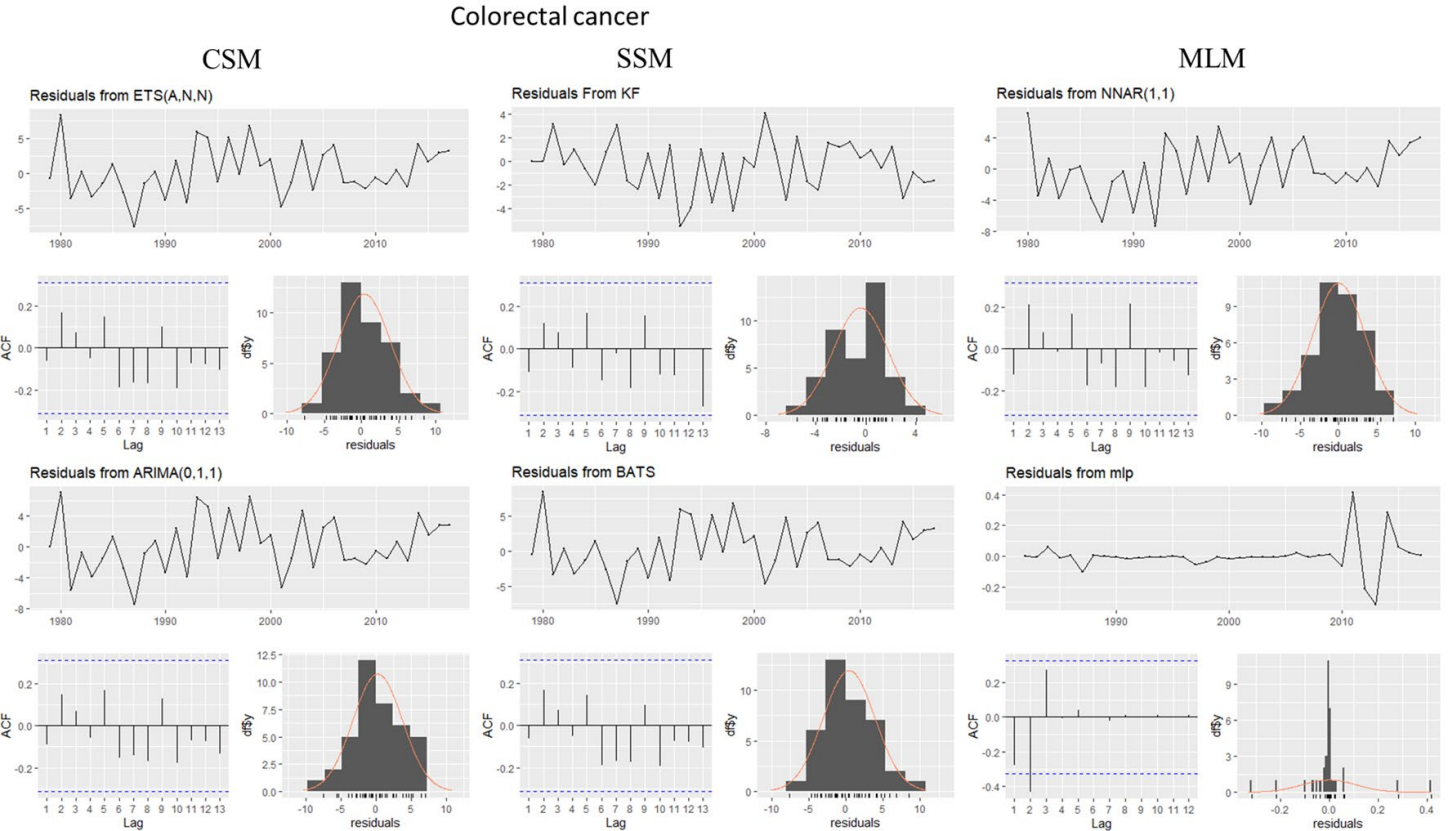
Colorectal cancer noise evaluation by model.

**Figure 7:**
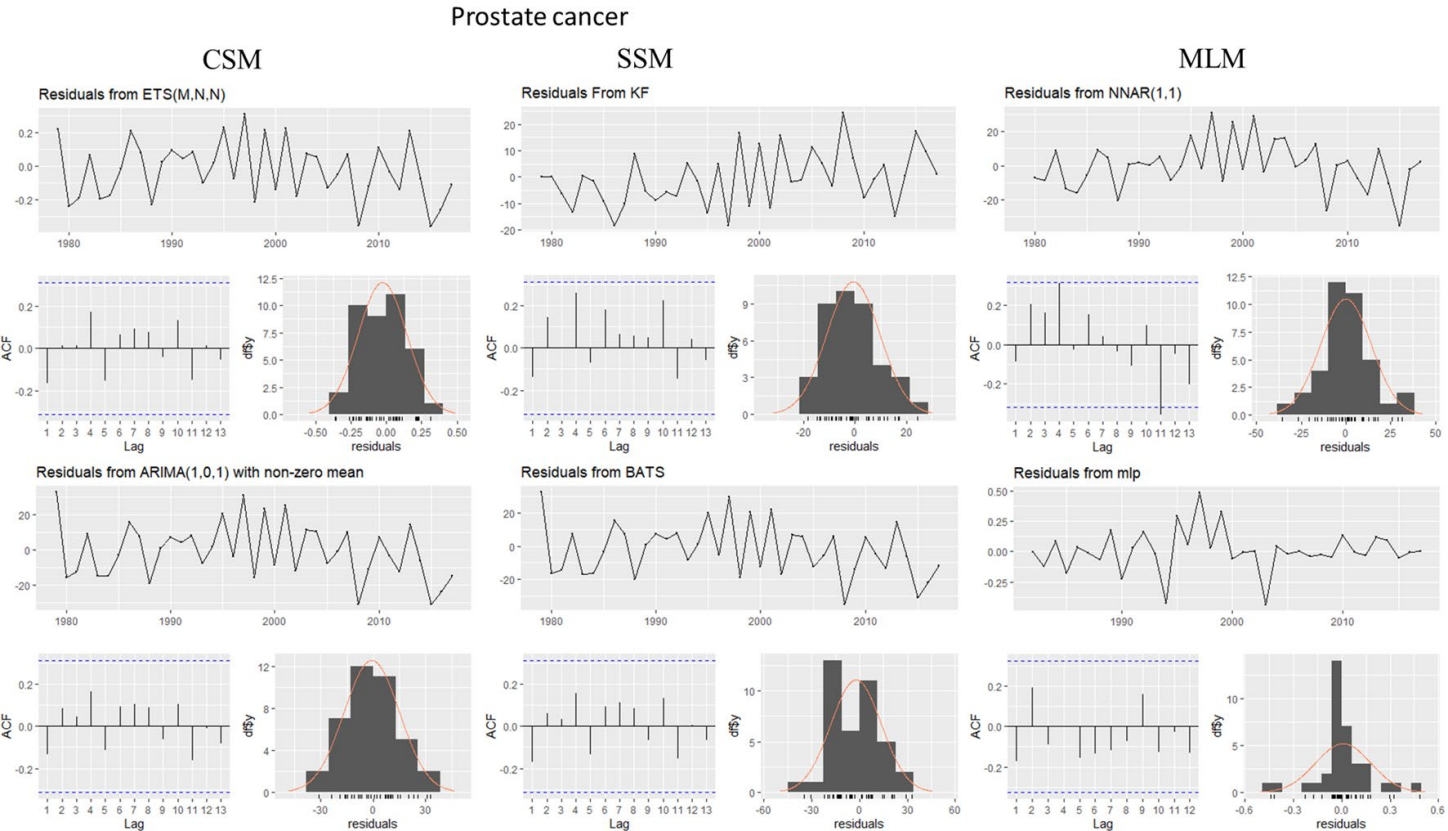
Prostate cancer noise evaluation by model.

**Figure 8:**
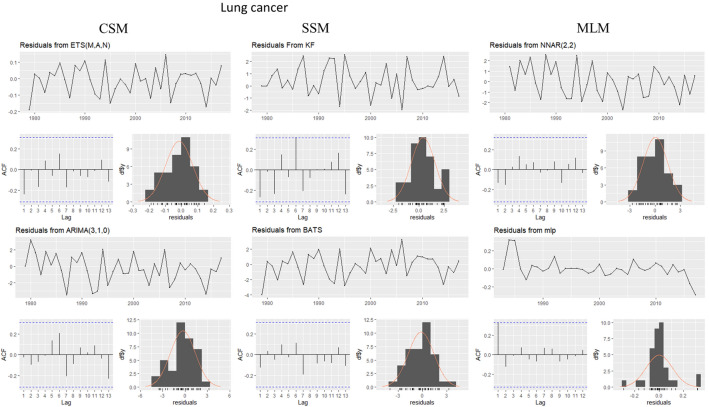
Lung cancer noise evaluation by model.

**Figure 9:**
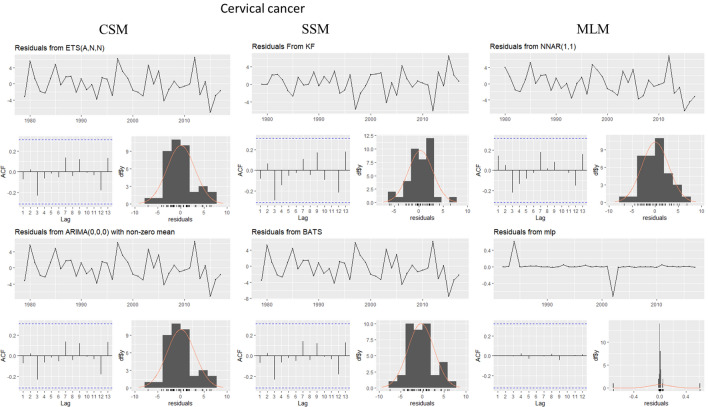
Cervical cancer noise evaluation by model.

**Figure 10:**
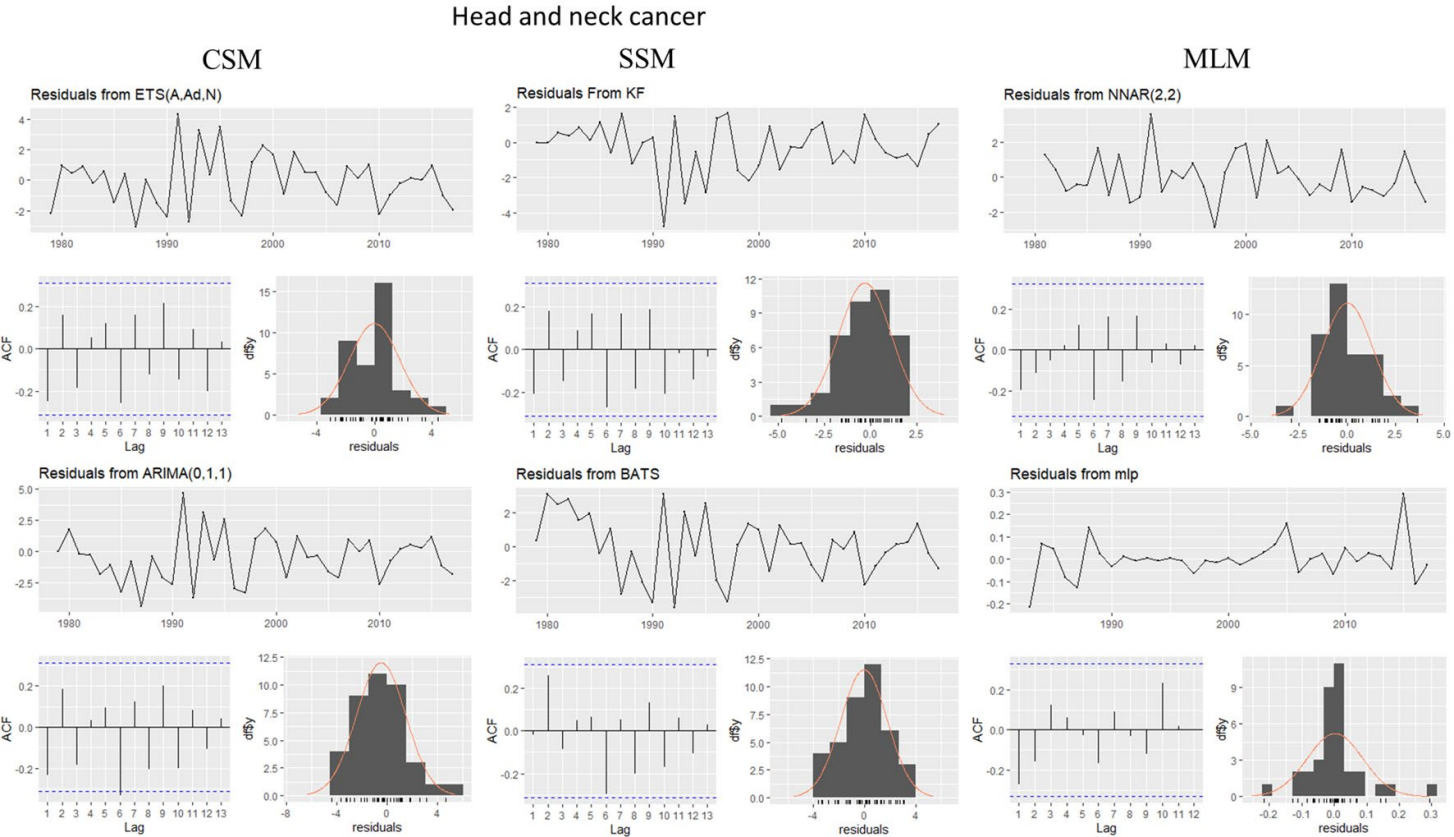
Head and Neck cancer noise evaluation by model.

**Figure 11:**
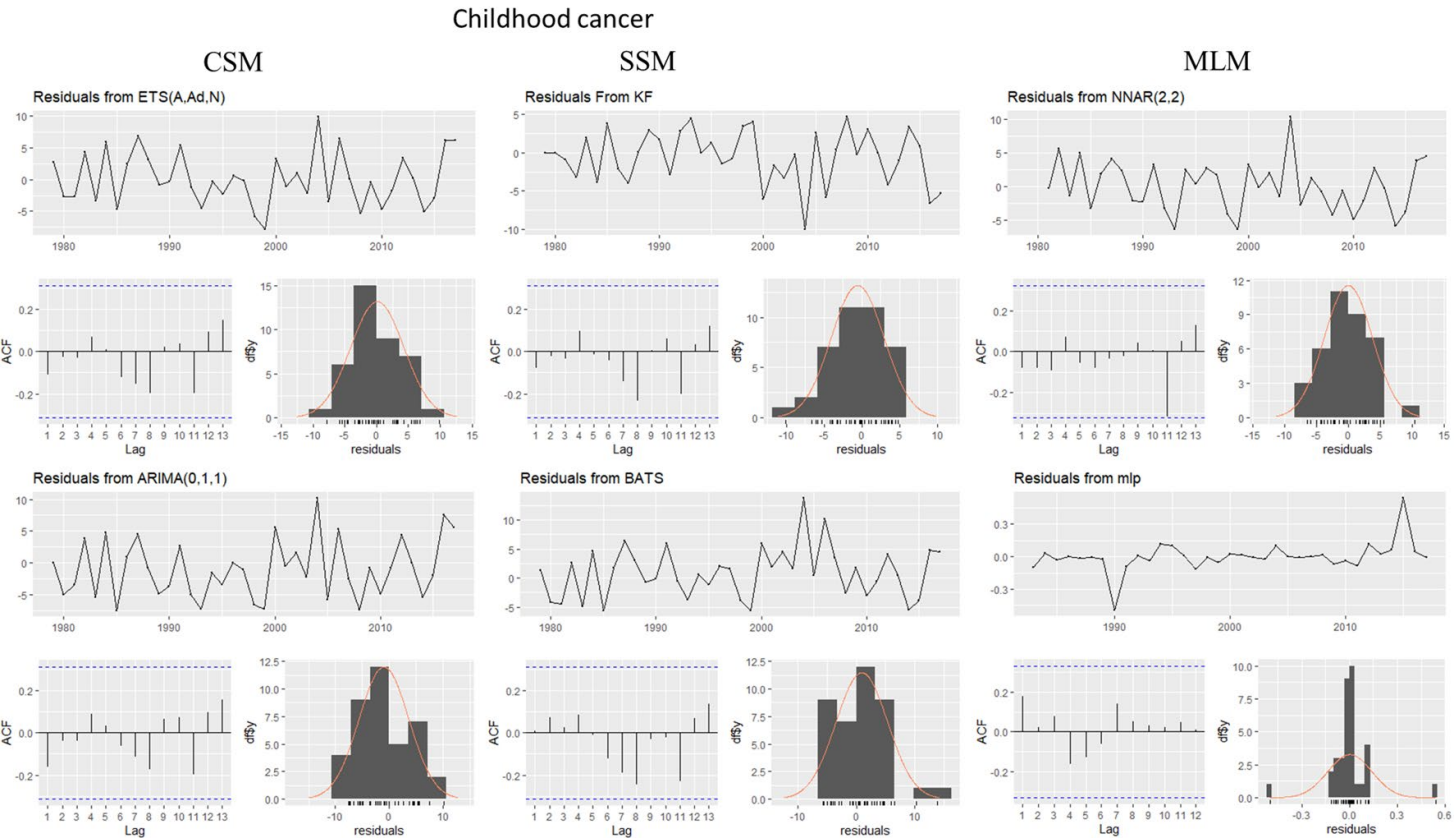
Childhood cancer noise evaluation by model.

The white noise failure evaluation by model and by cancer type is summarized in Table 10.

**Table 10:** White noise failure evaluation summary per type of cancer per model.

Failures	Breast	Colorectal	Prostate	Lung	Cervical	Head and neck	Childhood
Current	ACF				ACF		
ETS	ACF						
ARIMA	ACF					ACF	
TBATS	ACF						
KF	ACF			ST, ACF		NT	
NNETAR		BPT	ACF				
MLP	NT	ACF, NT, BPT	NT	NT, ACF	NT	NT	NT

Considering the criteria presented in Section Forecasting models evaluation to ensure an unbiased model, we must select the best model to each type of cancer evaluated discarding the result of the following failed (biased) models for:

- Current model, ETS, ARIMA, TBATS and KF to breast cancer which failed in Auto-correlation function (ACF) plot presented in Fig.  5 and, in normality test, MLP failed.
- MLP to colorectal cancer which failed in ACF plot presented in Fig.  6, Breusch-Pagan test and in normality test. NNETAR also failed in Breusch-Pagan test.
- NNETAR to prostate cancer which failed in Auto-correlation function (ACF) plot presented in Fig.  7 and MLP failed in normality test.
- KF to lung cancer which failed in student test, ACF plot presented in Fig.  8 and, in normality test and ACF plot, MLP failed.
- Cervical cancer presented residuals produced a significant ACF plot only to current model as presented in Fig.  9. MLP failed in normality test.
- ARIMA to head and neck cancer which failed in ACF plot presented in Fig.  10 and, in normality test, KF and MLP failed.
- MLP to childhood cancer which failed in normality test.

Thus, the best model to each cancer type are: NNETAR for breast, KF for colorectal, ARIMA for prostate, TBATS for lung, KF for cervical, the current method for Head and neck and KF for childhood.

**Figure 12:**
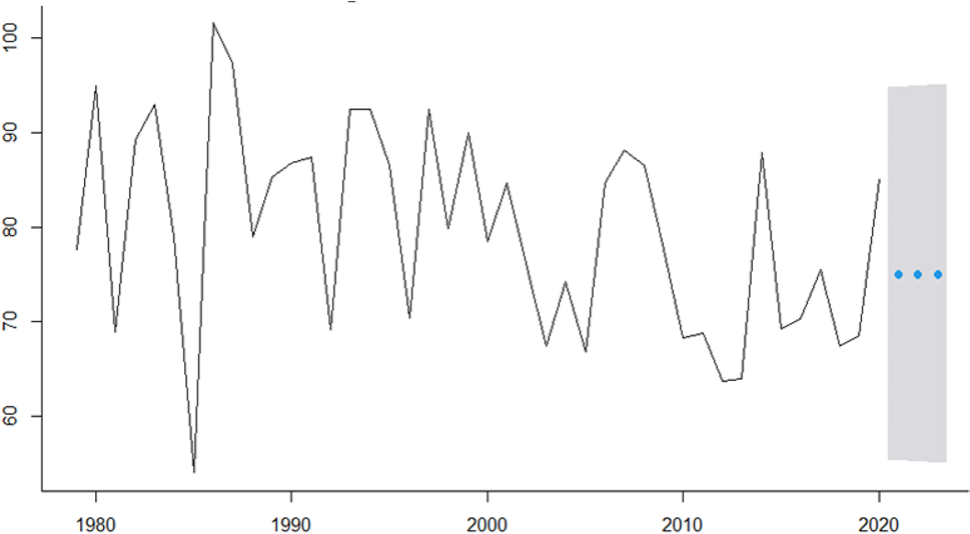
NNAR breast cancer *IRa* prediction values.

**Figure 13:**
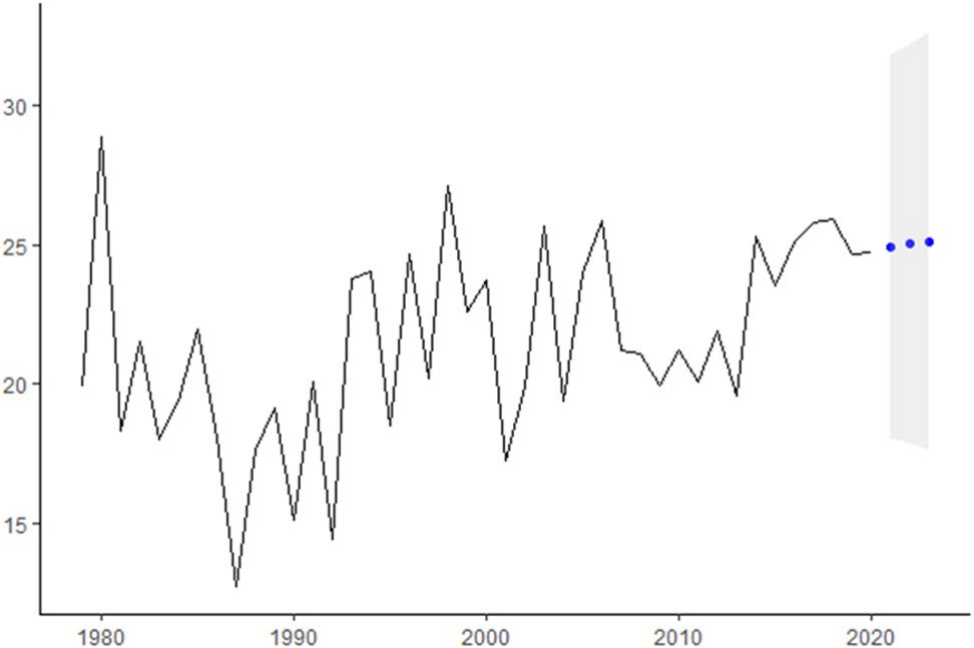
KF colorectal cancer *IRa* fitted and prediction values.

**Figure 14:**
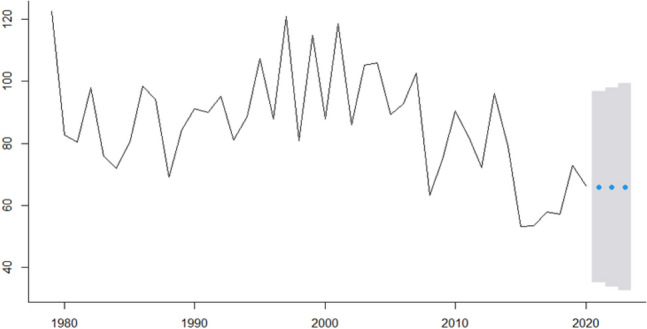
ARIMA prostate cancer *IRa* fitted and prediction values.

**Figure 15:**
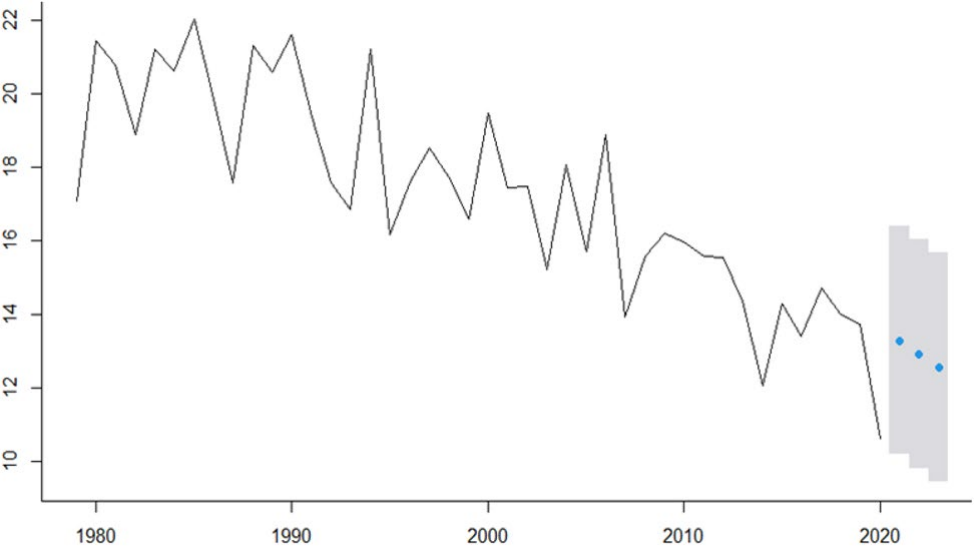
TBATS lung cancer *IRa* fitted and prediction values.

**Figure 16:**
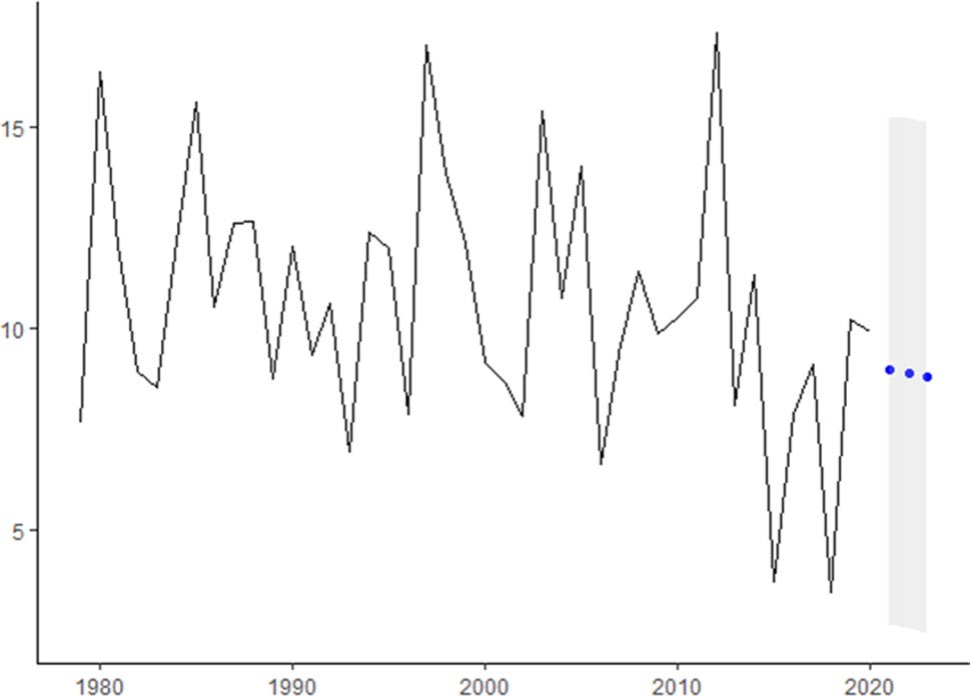
KF cervical cancer *IRa* fitted and prediction.

**Figure 17:**
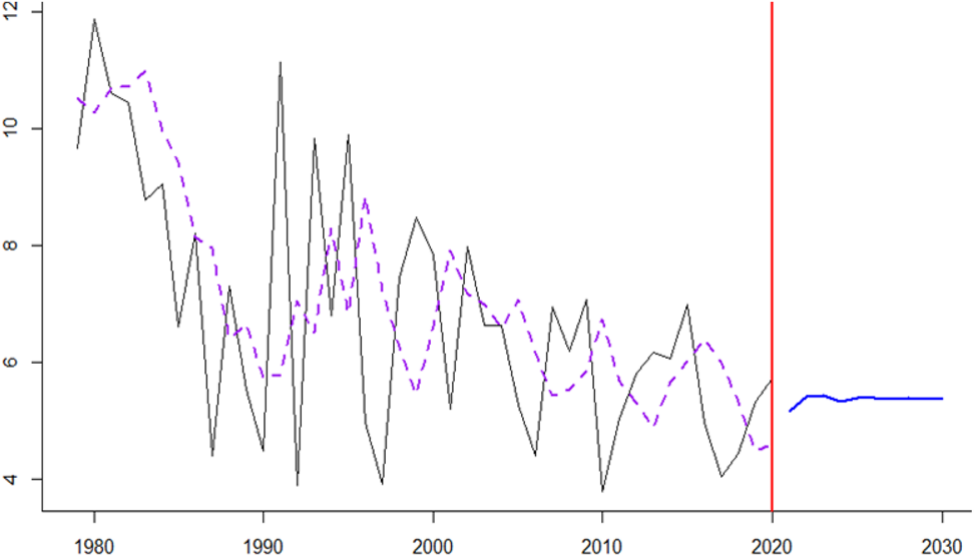
Current method head and neck cancer *IRa* fitted and prediction values.

**Figure 18:**
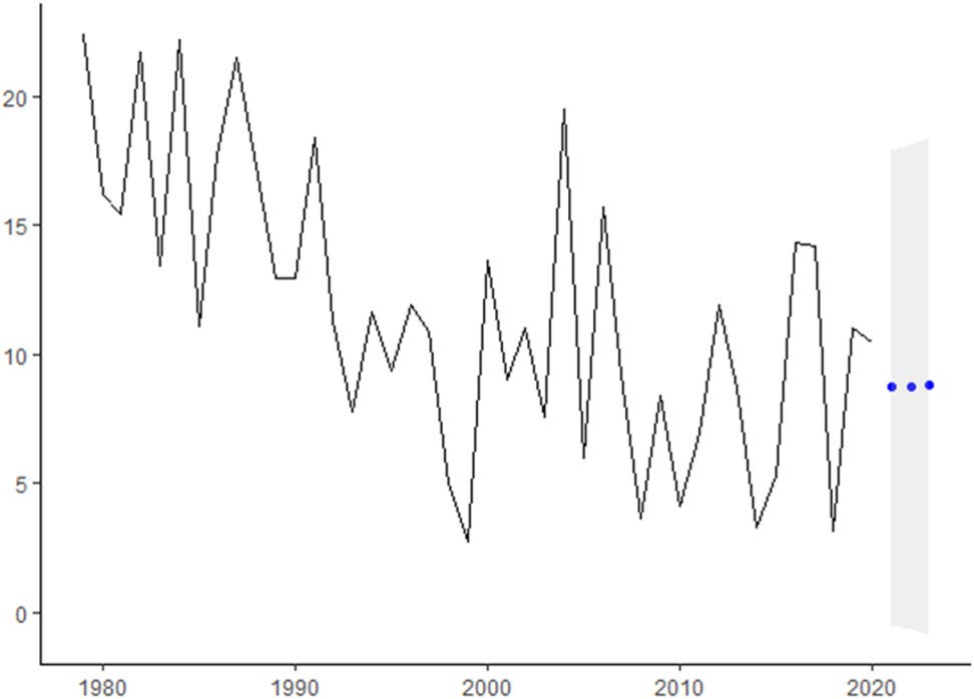
KF Childhood cancer *IRa* fitted and prediction values.

Their prediction plots can be seen respectively in Figs. 12, 13, 14, 15, 16, 17 and 18. The 3-year ahead prediction values are summarized in Table 11

**Table 11:** Three years *IRa* prediction using the best model to each cancer type.

Cancer Type	Best model	2021	2022	2023
Breast	NNETAR	79.158	79.158	79.158
Colorectal	KF	24.950	25.042	25.133
Prostate	ARIMA	65.891	65.891	65.891
Lung	TBATS	12.550	12.921	13.293
Cervical	KF	8.952	8.867	8.783
Head and Neck	Current	5.835	5.740	5.766
Childhood	KF	8.716	8.751	8.786

## Discussion

A limitation of this research could be observed in the method used to obtain the incidence of cancer in Brazil. This occurs because, in practice, the incidence is not measured. Thus, we used cancer incidence estimation methodologies proposed in Black et al.^23^, Ferlay et al.^24^ and Ferlay et al.^25^ which are based on the mortality rate discussed in Section Data collection.

Considering that the presented methodologies can give us the best cancer incidence estimation evaluating only time-series univariate models, our findings in Table 6 seem to indicate that the current model applied by INCA in Brazil to forecasting cancer incidence underperform in 6 of the 7 type of cancers proposed in this research. So, the presented methodologies seem to behave more adequately than the Brazilian’s current methodology.

It is important to note that we are working with the same type and amount of data that is used today, meaning that it would not be necessary to collect new variables in order to increase the accuracy of the forecast.

In addition, we did not see the CSM models outperform the others in any type of cancer, although ARIMA models (CSM) are the most widely used models in the current literature so far as we presented in Table 1.

These facts imply that, while there is no broad and reliable Population-Based Cancer Registries in the country, all research that use these data as a primary source will be limited; including this one.

However, it is necessary to consider that Brazil has continental dimensions and a technological backwardness that do not facilitate the implementation of this type of record. Although restrictive, the fact has not prevented research and public policies aimed to cancer prevention and control in the country, that surely could be more effective.

In this sense, we reinforce that it is not possible to invalidate what has been done in the country, but to plead for the opening of space so that new, more accurate forecast models can be adopted, aiming at supporting strategic decisions to face cancer in the country. Even because the current literature has used models that go in the opposite direction of the results presented by this research in Table 1.

For instance, MLM models were only used in Soltani et al.^14^ and Alrobai and Jilani^15^ works and only LTSM were evaluated. Considering SSM, the current literature presents only Lee et al.^10^ research in which only KF approach is proposed.

In Table 11, we see that SSM (KF and TBATS) was selected in four of seven type of cancers evaluated while MLM (NNETAR), CSM (ARIMA) and current method where selected to one type of cancer.

The evaluation process adopted in this research and presented in Section Forecasting models evaluation was crucial to identify and discard biased models to each type of cancer. If we had only considered in sample RMSE criterion (measuring the best fitted model, on average) to select the models to each type of cancer, MLP would be selected in all time-series evaluated.

On the other hand, if we considered only out of sample RMSE criterion (measuring the best predicted values, on average), ARIMA and MLP would be selected in two types of cancer while ETS, TBATS and KF would be selected in only one type of cancer time-series (NNETAR and current method would not be selected).

The noise evaluation process adopted also allowed us to state that the current model can potentially provide a biased prediction because it failed in ACF plot to Breast and Cervical cancer as we can see in Fig.  4. Therefore, we cannot classify it as statistically valid for making predictions.

It is important to note that both cancers affects the female population and keep using the current method could jeopardize efficient planning of resources for diagnosis and treatment for them.

Considering that, in Brazil, government policies and programs are mostly focused on these types of cancer the situation may pose an important challenge to be overcome.

Finally, by evaluating Brazilian’s current approach, CSM, SSM and MLM using four exclusion criteria (mean 0, normality, ACF and homoscedasticity tests) and one decision criteria (lowest out of sample RMSE) we were able to establish the best unbiased model to each type of cancer, as we wanted to illustrate. We also emphasize that by comparing different methods we can potentially improve the main issue addressed in this research: how to provide an unbiased and reliable cancer forecasting.

Although it is not the focus of this research, causal and multivariate time-series models associated with other control variables such as cigarette smoking as a predictor of lung cancer and HPV vaccination coverage for cervical cancer should be investigated. Another promising direction is to investigate age-period-cohort (APC) models and combine them with the time-series models proposed in this research.

## Conclusions

This research aimed to present and apply the main time-series-based models available in forecasting literature to the seven most prevalent types of cancer in Brazil. These models fall into three classes: classical statistical models, State-Space models, and machine learning models.

As mentioned in Theoretical Background section, it is the first attempt to apply unseen methods (TBATS, NNETAR and MLP) and the three classes of models to cancer prediction.

In Brazil, the incidence of cancer is not directly measured and must be estimated based on the mortality rate. Despite the challenge of not directly measuring cancer incidence, it is crucial for public health systems to estimate the incidence of a disease that ranks second in terms of mortality rate per 100,000 inhabitants.

While acknowledging the issue of not directly measuring incidence, our research mitigates this concern by utilizing the same data and employing the same cancer incidence estimation methods. This consistency ensures that our comparison between Brazil’s current prediction method and our proposed methods remains valid.

We also contributed to fulfill a literature gap identified in Table 1 by applying TBATS, MLP and NNETAR forecasting techniques predict seven cancer types in a Brazilian district.

Furthermore, we did not find any similar studies that compared the results of three classes of univariate time-series forecasting models or addressed more than one type of cancer.

When comparing only the error results (RMSE in sample and out of sample) between the approaches mentioned above and the current technique, we demonstrated that the current method underperforms for all types of cancer tested.

Moreover, in the Discussion section, we illustrated that, for breast and cervical cancers, the current approach applied in Brazil produced biased residuals, potentially affecting the quality and reliability of cancer incidence predictions in this country. Consequently, it may provide inaccurate information to healthcare decision-makers.

Therefore, we suggest that the methods evaluated in this study should be integrated into Brazil’s cancer forecast methodology to provide a reliable prediction for healthcare decision-makers.

To further researches, we also suggest a comparison between MLM time-series approaches. NNETAR and MLP (covered in this research) with LTSM which had been also used in recent previous works like Soltani et al.^14^ and Alrobai and Jilani^15^ presented in Table 1.

Although it was not the focus of this research, it should be noted that age-period-cohort (APC), previously mentioned in Section Theoretical Background, and Ensemble APC analysis as well as considering the birth-cohort effects^39,40^ have potential to provide more accurate forecasts compared to traditional time-series methods that only consider period components.

Finally, by contributing with a proposal for the application of a set of tested forecasting methods to estimate the incidence of cancer in Brazil, it is intended that the results encourage a discussion on the adoption of anticipatory actions, aimed at prevention and the provision of means and resources for the early detection of the most prevalent types of cancer.

In this sense, to provide more robust predictions causal models could be also taking into account like we can see in^41–47^ applied to other diseases. Using them it is possible to evaluate the impact of smoking reduction or HPV vaccines strategies for lung and cervical cancer respectively, for instance.

### Supplementary Information

Supplementary Information 1.

Supplementary Information 2.

## Data Availability

All relevant data are within the manuscript and its Supporting Information files.

## Code availability

At Supplementary Material (Forecasting code.R).
